# Chinese Pediatrician Attitudes and Practices Regarding Child Exposure to Secondhand Smoke (SHS) and Clinical Efforts against SHS Exposure

**DOI:** 10.3390/ijerph120505013

**Published:** 2015-05-08

**Authors:** Kaiyong Huang, Abu S. Abdullah, Haiying Huo, Jing Liao, Li Yang, Zhiyong Zhang, Hailian Chen, Guangmin Nong, Jonathan P. Winickoff

**Affiliations:** 1 School of Public Health, Guangxi Medical University, Nanning, Guangxi 530021, China; E-Mails: huangka0319@sina.com (K.H.); huohy0117@126.com (H.H.); rpazz@163.com (Z.Z.); chlq869007621@163.com (H.C.); 2 Global Health Program, Duke Kunshan University, Kunshan, Jiangsu 215347, China; 3 Duke Global Health Institute, Duke University, Durham, NC 27708, USA; 4 School of Medicine, Boston Medical Center, Boston University, Boston, MA 02118, USA; 5 Department of Pediatrics, The First Affiliated Hospital of Guangxi Medical University, Nanning, Guangxi 530021, China; E-Mails: gxlmd@126.com (J.L.); ngm8525@hotmail.com (G.N.); 6 Massachusetts General Hospital (MGH) Center for Child and Adolescent Health Research and Policy, Harvard Medical School, Boston, MA 02114, USA; E-Mail: jwinickoff@partners.org

**Keywords:** pediatrician, secondhand smoke, exposure, smoking cessation

## Abstract

Background: Secondhand Smoke (SHS) exposure is a leading cause of childhood illness and premature death. Pediatricians play an important role in helping parents to quit smoking and reducing children’s SHS exposure. This study examined Chinese pediatricians’ attitudes and practices regarding children’s exposure to SHS and clinical efforts against SHS exposure. Methods: A cross-sectional survey of pediatricians was conducted in thirteen conveniently selected hospitals in southern China, during September to December 2013. Five hundred and four pediatricians completed self-administered questionnaires with a response rate of 92%. *χ^2^* tests were used to compare categorical variables differences between smokers and non-smokers and other categorical variables. Results: Pediatricians thought that the key barriers to encouraging parents to quit smoking were: lack of professional training (94%), lack of time (84%), resistance to discussions about smoking (77%). 94% of the pediatricians agreed that smoking in enclosed public places should be prohibited and more than 70% agreed that smoking should not be allowed in any indoor places and in cars. Most of the pediatricians thought that their current knowledge on helping people to quit smoking and SHS exposure reduction counseling was insufficient. Conclusions: Many Chinese pediatricians did not have adequate knowledge about smoking and SHS, and many lacked confidence about giving cessation or SHS exposure reduction counseling to smoking parents. Lack of professional training and time were the most important barriers to help parents quit smoking among the Chinese pediatricians. Intensified efforts are called for to provide the necessary professional training and increase pediatricians’ participation in the training.

## 1. Introduction

The tobacco epidemic is one of the biggest public health threats the World is facing, killing approximately 6 million people each year. More than 5 million of these deaths are as a result of direct tobacco use, while more than 600,000 are attributable to Secondhand Smoke (SHS) exposure [1]. 80% of the deaths occur in low- and middle-income countries, and the death toll could rise to more than 8 million per year by 2030 if the current trend of increasing global tobacco use remains unaddressed [1–3]. China, a middle-income country, is the World’s largest producer and consumer of tobacco, with 350 million smokers [4], and has almost 740 million non-smokers passively exposed to SHS, including 180 million children under 15 years old [5,6]. Nearly 100,000 deaths related to SHS exposure occur in China annually [5]. There is no known safe level of SHS exposure [7]. It is reported that children exposed to SHS have higher rates of lower respiratory illness, asthma and asthma exacerbations, coughs, middle ear infections, hospitalizations, cardiovascular disease and sudden infant death syndrome [8,9].

Smoking bans were implemented in public areas including workplaces, government buildings, public transportation, healthcare facilities, supermarkets, schools, bars and restaurants in 2010 to reduce the prevalence of SHS exposure in China [5]. However, SHS exposure at home which is often neglected is the primary source of exposure to tobacco smoke for children [10,11]. In 2010, the prevalence of SHS exposure at home in China was 67.3% [5], much higher than the United States [12], Mexico and India [13].

Smoking cessation is a priority for reducing the harm and burden caused by smoking-attributable diseases [14,15]. There is the modeling effect on children whose parents smoke at home, and parental smoking is a potent indicator of which children will become adult smokers [16]. Among the physicians group, pediatricians can address both SHS exposure to children and parental smoking cessation and therefore are in a critical position to implement parental tobacco control programs. Pediatricians believe that exposure to SHS is harmful to children’s health and have expressed opposition to SHS exposure among children [17,18]. In the United States, pediatricians are encouraged by the U.S. Surgeon General and the American Academy of Pediatrics (AAP) to directly address SHS with parents and provide guidance on smoking cessation [19].

In many studies on physicians, beliefs regarding the effectiveness of counseling and medications for smoking cessation and reducing SHS are different. For example, a systematic review indicated that the majority of general practitioners and family physicians thought discussing smoking cessation with their patients were ineffective [20]. Another systematic review showed that when physicians provided smoking cessation advice for patients it significantly increased the odds of quitting smoking [21]. However, pediatricians have yet to fully embrace their role in helping parents quit smoking and helping children reduce SHS exposure [19]. No study has been conducted on pediatricians’ attitudes and practices about children SHS exposure in China. To explore Chinese pediatricians’ attitudes and practices regarding children exposure to SHS and clinical effort against SHS exposure, we conducted a quantitative study on pediatricians residing in southern China.

## 2. Methods

### 2.1. Design and Sample

A cross-sectional survey of pediatricians was conducted from September to December 2013 to assess Chinese pediatricians’ attitudes and practices regarding children exposure to SHS and clinical efforts against SHS exposure. A standardized Chinese language self-administered questionnaire was used to collect data from 550 pediatricians working in the thirteen hospitals—twelve grade Ⅲ and one grade (I)—selected conveniently in four major cities (Nanning, Liuzhou, Guilin and Qinzhou) of Guangxi Province (a southern Chinese province), the People’s Republic of China. Questionnaires were distributed to each pediatrician working in the selected hospitals by the directors. Pediatricians were requested to complete all the questions on the questionnaire, seal the questionnaire in an envelope and drop it in a designated box kept in the general office of the department. Our study coordinator then collected the sealed questionnaires from each of the directors and checked all questionnaires. If questionnaires had any unclear answers, unfinished questions, and/or logistic errors, our study coordinator would contact the individual pediatrician by telephone or interview them face to face. To compensate for their time, each participant was given a cash amount of RMB 100 ($15). Our study protocols were approved by the Ethical Committee of Guangxi Medical University and we received informed consent from all individuals who agreed to participate in the study.

### 2.2. Measures

The questionnaire was developed with reference to the questionnaires previously used by the investigators team in the United States [22] and in China [23]. The questionnaire included questions on the following domains: demographic and other characteristics, barriers to smoking cessation counseling, attitudes and practices toward tobacco use and SHS exposure, and knowledge of SHS exposure.

We collected demographic and other information on gender, age, physician type, number of years studied at medical school, smoking behavior (smoker, non-smoker), and other questions on “Have you ever received any formal training in smoking cessation?” (Yes or No), “Have you read China’s smoking cessation guidelines?” (Yes, No, and Never heard about it), “Have you read international smoking cessation guidelines?” (Yes, No, and Never heard about it). We defined “smoker” as those who smoked at the time of the survey, and defined “non-smoker” as those who never smoked or quitted smoking at the time of survey [11]. Pediatricians’ perceived barriers to the delivery of smoking cessation counseling to smoking parents of children were assessed by asking seven questions (Table S1), with response categories of “Major Barrier, Moderate Barrier, Minor Barrier and Not a Barrier”. For analysis, responses were condensed to two categories of “Major Barrier/Moderate Barrier”, and “Minor Barrier/Not a Barrier”. Questions asked in other domains of the questionnaire and the related response categories are available in Table S1. 

### 2.3. Analyses

Two members of the research team coded each questionnaire and entered all data with Epidata 3.1, and then made a data consistency check. *χ^2^* tests were used to compare categorical variables differences between smoker and non-smoker. A *p*-value of <0.05 (two-tailed) was considered statistically significant. We used SPSS version 13.0 to conduct all statistical analyses.

## 3. Results

### 3.1. Demographic and Other Characteristics Information

A total of 504 participants successfully completed the questionnaires, with a response rate of 92% (504/550). Of the respondents, 64% were female with 74% being between the ages of 20 and 40, and 75% were resident and attending physicians, 77% of the participants had received 5 years of education in medical school and 17% smoked at the time of the survey. 81% of the samples hadn’t received formal training in smoking cessation, and 64% of all pediatricians hadn’t read China’s smoking cessation guidelines, with 21% having never heard about them. Three hundred and fifty nine participants (71%) hadn’t read any international smoking cessation guidelines, and 22% had never heard about them (Table 1).

**Table 1:** Demographic and other characteristics of pediatricians, Guangxi, China 2013 (n = 504).

Variables	N	%
***Gender***		
Male	182	36
Female	322	64
***Age***		
20–30	215	43
31–40	159	31
41–50	89	18
Above 50	41	8
***Physician type***		
Resident Physician	223	45
Attending Physician	151	30
Associate Chief Physician	88	17
Chief Physician	42	8
***Number of years studied at medical school***		
5 Years	388	77
More than 5 years	116	23
***Smoking status***		
Current smoker	82	16
Non-smoker	422	84
***Received formal training in smoking cessation***		
No	399	81
Yes	96	19
***Have read China’s smoking cessation guidelines***		
No	322	64
Yes	77	15
Never heard about it	105	21
***Have read international smoking cessation guidelines***		
No	359	71
Yes	36	7
Never heard about it	109	22

### 3.2. Barriers to Smoking Cessation

Most of the pediatricians perceived the key barriers to be: “Parents are resistant to discuss about smoking” (77%), “It is hard to find a time to talk with parents” (84%), “Lack of professional training in the area of tobacco cessation counseling” (94%), “Lack of a standard of care requiring pediatricians to provide smoking cessation or SHS exposure reduction intervention” (64%), “It is hard to make system changes that would support parental smoking cessation at our hospital” (76%), “Not convinced that advice and/or available therapies would work” (61%) were the barriers to the delivery of smoking cessation counseling to smoking parents of children (as shown in Table 2). However, when comparing the differences of all the perceived barriers between smokers and non-smokers, “Lack of professional training in the area of tobacco cessation counseling” was the only variable that showed a significant difference (*p* < 0.001). 98% of the non-smokers compared to 65% of smokers perceived that lack of professional training was a barrier to provide smoking cessation service to children’s smoking parents (Table 2).

**Table 2:** Pediatricians’ perceived barriers to the delivery of smoking cessation counseling to smoking parents of children by smoking status.

Variables	All Respondents	Smoker	Non-Smoker	*p-*Value
	(N = 504)	(N1 = 82)	(N2 = 422)	
	Major/Moderate	Major/Moderate	Major/Moderate	
	Barrier	Barrier	Barrier	
	N (%)	N1 (%)	N2 (%)	
Parents are resistant to discussion about smoking	390 (77)	62 (76)	328 (78)	0.675
It is hard to find a time to talk with parents	422 (84)	65 (79)	357 (84)	0.232
Lack of professional training in the area of tobacco cessation counseling	455 (94) *	40 (65) *	415 (98) *	<0.001
Lack of a standard of care requiring pediatricians to provide smoking cessation or SHS exposure reduction intervention	321 (64)	53 (65)	268 (64)	0.846
Lack of insurance coverage for smoking cessation medication	279 (55)	43 (52)	236 (56)	0.561
It is hard to make system changes that would support parental smoking cessation at our hospital	383 (76)	63 (77)	320 (76)	0.846
Not convinced that advice and/or available therapies would work	306 (61)	53 (65)	253 (60)	0.427

* Due to the missing values in some variables, the total number of responses for the variable “Lack of professional training in the area of tobacco cessation counseling” was 482 (61 smokers and 421 non-smokers).

### 3.3. Attitudes and Practices

Table 3 provides results for the pediatricians’ attitudes and practices regarding children’s exposure to SHS and clinical efforts against SHS exposure. Most of the respondents have good knowledge about SHS. Compared to the pediatricians who were smokers, the non-smokers were more likely to agree that SHS causes children’s pulmonary disease (*p* = 0.028), SHS causes asthma in children (*p* = 0.031), and paternal smoking increases the risk of lower respiratory tract illnesses such as pneumonia in exposed children (*p* < 0.001).

Of the 504 pediatricians, 94% expressed the opinion that smoking in enclosed public places should be prohibited. In addition, more than 70% of the participants agreed smoking should not be allowed in any indoor room and in cars (non-smokers were more likely to agree to these issues (*p* < 0.001; *p* < 0.001)) (Table 3).

More than 79% of the respondents agreed that pediatricians should routinely ask their patients’ parents about their smoking habits and advise the smoking parents to quit smoking (non-smokers were more likely to agree (*p* < 0.001, and *p* < 0.05). 56% of the participants thought pediatricians can help patients’ parents stop smoking, 60% believed physician counseling for smoking cessation was effective, and 53% believed pharmacological products were effective in helping people quit smoking. However, 51% expressed the opinion that smoking cessation counseling is not an efficient use of their time. Nearly 93% agree that physicians should advise parents to avoid smoking around children and that they shouldn’t smoke in front of their patients.

On average, 51% of the pediatricians admitted they could find resources to help patient’s parents to quit smoking in their hospital, but 62% didn’t know the best strategies for helping patients’ parents to stop smoking. In addition, only 17% of the pediatricians thought their current knowledge was sufficient for helping patients’ parents to stop smoking, and 30% thought their current knowledge was sufficient for helping parents to reduce children’s’ SHS exposure. More than 61% were not familiar with the guidelines for smoking cessation, and only 16% could assess a smoker’s different stages of readiness to quit.

**Table 3:** Attitudes and practices regarding child exposure to SHS and clinical effort against SHS exposure by smoking status.

Variables	All Respondents	Smoker	Non-Smoker	*p-*Value
	(N = 504)	(N1 = 82)	(N2 = 422)	
	Agree	Agree	Agree	
	N (%)	N1 (%)	N2 (%)	
***Knowledge about SHS***				
SHS causes Sudden Infant Death Syndrome	336 (67)	59 (72)	277 (66)	0.267
SHS causes adult lung cancer	487 (97)	78 (95)	409 (97)	0.681
SHS causes adult heart disease	392 (78)	64 (78)	328 (78)	0.949
SHS causes bronchitis	474 (94)	77 (94)	397 (94)	0.952
SHS causes children’s pulmonary disease	491 (97)	77 (94)	414 (98)	0.028
SHS causes asthma in children	487 (97)	76 (93)	411 (97)	0.031
SHS causes respiratory infections in children	465 (92)	73 (89)	392 (93)	0.230
Breathing air in a room today where people smoked yesterday can harm the health of infants and children	368 (73)	60 (73)	308 (73)	0.972
Paternal smoking increases the risk of lower respiratory tract illnesses such as pneumonia in exposed children	458 (91)	62 (76)	396 (94)	<0.001
***Support for smoking bans***				
Smoking in enclosed public places should be prohibited	476 (94)	75 (91)	401 (95)	0.198
Smoking is not allowed in any indoor room	354 (70)	42 (51)	312 (74)	<0.001
Smoking is not allowed in car	441 (88)	61 (74)	380 (90)	<0.001
***Support for clinical effort against SHS exposure***				
Pediatricians can help patients’ parents to stop smoking	282 (56)	47 (57)	235 (56)	0.786
Smoking cessation counseling is not an efficient use of my time	256 (51)	47 (57)	209 (50)	0.197
Beliefs regarding effectiveness of physician counseling for smoking cessation	304 (60)	51 (62)	253 (60)	0.704
It is easy for me to find resources in my hospital to help my patient’s parents to quit smoking	256 (51)	48 (58)	208 (49)	0.125
Physicians should not smoke in front of their patients	471 (93)	74 (90)	397 (94)	0.199
Advise patients who smoke to avoid smoking around children	469 (93)	74 (90)	395 (94)	0.274
Routinely ask about their patients smoking habits	425 (84)	54 (66)	371 (88)	<0.001
Routinely advise their smoking patients to quit smoking	398 (79)	57 (69)	341 (81)	0.022
***Attitudes towards counseling and treatment***				
I am not familiar with the guidelines for stop smoking	309 (61)	49 (60)	260 (62)	0.752
I am unaware of the best strategies for helping my patients’ parents to stop smoking	313 (62)	51 (62)	262 (62)	0.985
Pharmacological products are effective in helping people quit smoking	266 (53)	42 (51)	224 (53)	0.757
My current knowledge is sufficient for helping parents to reduce SHS exposure to children	151 (30)	23 (28)	128 (30)	0.680
My current knowledge is sufficient for helping patients to stop smoking	87 (17)	15 (18)	72 (17)	0.787
I can assess a smoker’s different stages of readiness to quit	82 (16)	12 (15)	70 (17)	0.661

## 4. Discussion

To the best of our knowledge, this cross-sectional survey is the first study on pediatrician attitudes and practices regarding child exposure to SHS and clinical efforts against SHS exposure in China. The findings suggest that pediatricians should participate in formal training on addressing parental smoking to reduce children’s SHS exposure from parental smoking. For example, our findings showed that 81% of the pediatricians didn’t receive formal training on smoking cessation, and 94% thought lack of professional training was a barrier to the delivery of smoking cessation counseling to smoking parents of children. Similar barriers have also been reported in other studies [17,19,24]. The lack of efficient and effective training might be one reason why pediatricians rarely implement effective children’s SHS exposure reduction interventions with smoking parents [24,25]. Similarly, other studies [17,19,24,26], have shown lack of time to talk with parents as another key barrier to reducing SHS exposure to children and promoting smoking cessation counseling. Researchers have indicated pediatrician’s provision of a brief advice regarding children’s exposure to SHS as an effective first step to make children’ parents quit smoking [27,28]. A national survey of pediatric nurses in the United States showed that parents’ resistance to discussions about smoking was one of the most common barriers to counseling parent [29], and we obtained a similar result in 77% of all the pediatricians.

Earlier research studies reported that physicians who were nonsmokers were more likely to support complete smoking ban at home [11,28], possess better knowledge about the hazards of smoking [23], provide smoking cessation advise [23,29,30], and believe that smoking cessation counseling is effective to promote smoking cessation [17,23]. In this study, most of the respondents had good knowledge about SHS, with non-smoking pediatricians reporting better results than smokers on certain issues. Possible explanations may be that the non-smoking pediatricians knew more about the dangers of smoking, SHS exposure, and health consequences of smoking to themselves and others, and, hence, refrained from smoking. This underscores the need to increase knowledge about the harms of smoking and SHS among the pediatricians. Most of the respondents supported smoking bans in enclosed public places, indoor rooms and cars, with the non-smoker more likely to agree to issues of “Smoking is not allowed in any indoor room” and “Smoking is not allowed in cars”. Consistent with the findings of an earlier study among Chinese physicians [29], smoker and non-smoker physicians did not differ significantly (*p* > 0.05) in their knowledge about the fact that SHS causes sudden infant death syndrome or SHS causes adult lung disease or heart disease. Besides examining the differences according to the smoking status, we have also examined pediatrician’s attitudes and practices by gender, age group, physician type and whether they had received any tobacco control training. However, we did not find any significant differences in these categorical analyses (data not shown).

Evidence suggests that counseling [31] and cessation medications [32,33] are effective for smoking cessation. Lately, a study also reported that providers’ self-confidence on tobacco intervention may improve the frequency of environmental tobacco smoke exposure related actions [19]. Our study found that over 50% of the pediatricians were confident that pediatricians can help patients’ parents to quit smoking, and believed physician counseling and pharmacological products were effective in helping smokers quit smoking, which was positive and useful information supporting the clinical efforts against SHS exposure. In our study, more than 79% of the pediatricians agreed that they should routinely ask their patients about their smoking habits and advise them to quit smoking, with the pediatricians who were non-smokers more likely agreeing to these issues compared to the smokers. However, for the reasons of lack of time, training, and resources, most of the pediatricians didn’t screen parents smoking status and gave quit smoking advise unless a parent’s child was being treated for respiratory diseases [17,19,31]. This is despite the fact that asking parents about their smoking habits and giving simple smoking cessation intervention would only take the pediatrician about 30 sec to 3 min [34]. The AAP also indicated that pediatricians should query about patients’ tobacco use and exposure, and help smoking parents to quit smoking [35].

Even though many interventions for quitting smoking have been implemented in the United States, 52% of pediatricians were unaware of the best strategies for helping parents to stop smoking and 51% reported that it was not easy to find resources in their community to help parents quit [36]. We obtained a similar result with 51% of the pediatricians saying they could find resources to help patient’s parents to quit smoking, but more than 60% were not familiar with the guidelines for cessation and unaware of the best strategies for stopping smoking. In an earlier study, pediatricians did not have adequate knowledge for helping quit smoking [37]. In our study, few of the pediatricians thought that their current knowledge was sufficient for helping parents to quit smoking (17%) and to reduce SHS exposure to children (30%), and only 16% could assess a smoker's different stages of readiness to quit. The insufficient knowledge will most likely be a key barrier to helping parents to quit smoking in China. It is therefore important to enhance the training and publicity about smoking cessation, and implement activities for helping parents to quit smoking. 

This study had several limitations. First, the sample may not be representative of the whole pediatrician population in China. Because all the selected hospitals were located in urban area of Guangxi Province, and most of the pediatricians come from grade Ⅲ hospitals (*i.e.*, the hospitals with best facilities, advanced technologies and resources), therefore, the findings may not be generalizable to other lower grade hospitals (*i.e.*, Grade 1 or Grade II). Secondly, nurses can play an important role in helping parents quit smoking and reduced children SHS exposure, but we didn’t investigate pediatric nurses in the current study. To make the findings more generalizable, a follow-up survey may select both pediatricians and nurses from all the three grades of hospitals. 

## 5. Conclusions

We found that over one-half of the pediatricians believed the fact that pediatricians can help children’s parents quit smoking and believed smoking cessation counseling was effective in helping people quit smoking. However, most of the pediatricians’ current knowledge was insufficient for reducing children SHS exposure and helping parents to quit smoking. Lack of professional training and time were the most important barriers to help parents stop smoking. We call for intensified efforts to carry out the needed professional training and increase pediatricians’ participation in the training, at the same time, asking about their patients smoking habits and advising their smoking patients to quit smoking routinely.

## Supplementary Files

Supplementary File 1
