# Supplementary material for: Chinese Pediatrician Attitudes and Practices Regarding Child Exposure to Secondhand Smoke (SHS) and Clinical Efforts against SHS Exposure
Source: Int J Environ Res Public Health. 2015 May 8;12(5):5013–25. doi: 10.3390/ijerph120505013 (PMC4454951; doi:10.3390/ijerph120505013)
Supplement: Supplementary File 1 [file ijerph-12-05013-s001.pdf]

Supplementary Information

Chinese Pediatrician Attitudes and Practices Regarding Child Exposure to Secondhand Smoke (SHS) and Clinical Effort against SHS Exposure

---

Table S1. Major questions included in the survey questionnaire and the responses categories.

Domains	Survey Questions	Response Categories
Pediatricians' perceived barriers	Parents are resistant to discussion about smoking	Major Barrier, Moderate Barrier, Minor Barrier and Not a Barrier
	It is hard to find a time to talk with parents	Major Barrier, Moderate Barrier, Minor Barrier and Not a Barrier
	Lack of professional training in the area of tobacco cessation counseling	Major Barrier, Moderate Barrier, Minor Barrier and Not a Barrier
	Lack of a standard of care requiring pediatricians to provide smoking cessation or SHS exposure reduction intervention	Major Barrier, Moderate Barrier, Minor Barrier and Not a Barrier
	Lack of insurance coverage for smoking cessation medication	Major Barrier, Moderate Barrier, Minor Barrier and Not a Barrier
	It is hard to make system changes that would support parental smoking cessation at our hospital	Major Barrier, Moderate Barrier, Minor Barrier and Not a Barrier
	Not convinced that advice and / or available therapies would work	Major Barrier, Moderate Barrier, Minor Barrier and Not a Barrier
Support for smoking bans	Smoking in enclosed public places should be prohibited	Strongly agree, Agree, Disagree and Strongly disagree
	Smoking is not allowed in any indoor room	Strongly agree, Agree, Disagree and Strongly disagree
	Smoking is not allowed in car	Strongly agree, Agree, Disagree and Strongly disagree
Support for clinical effort against SHS exposure	Pediatricians can help patients' parents to stop smoking	Strongly agree, Agree, Disagree and Strongly disagree
	Smoking cessation counseling is not an efficient use of my time	Strongly agree, Agree, Disagree and Strongly disagree
	Beliefs regarding effectiveness of physician counseling for smoking cessation	Strongly agree, Agree, Disagree and Strongly disagree
	It is easy for me to find resources in my hospital to help my patient's parents to quit smoking	Strongly agree, Agree, Disagree and Strongly disagree
	Physicians should not smoke in front of their patients	Strongly agree, Agree, Disagree and Strongly disagree

Table S1. *Cont.*

Domains	Survey Questions	Response Categories
	Physicians should advise patients who smoke to avoid smoking around children	Strongly agree, Agree, Disagree and Strongly disagree
	Physicians should routinely ask about their patients smoking habits	Strongly agree, Agree, Disagree and Strongly disagree
	Physicians should routinely advise their smoking patients to quit smoking	Strongly agree, Agree, Disagree and Strongly disagree
Attitudes towards counseling and treatment	I am not familiar with the guidelines for stop smoking	Strongly agree, Agree, Disagree and Strongly disagree
	I am unaware of the best strategies for helping my patients' parents to stop smoking	Strongly agree, Agree, Disagree and Strongly disagree
	Pharmacological products are effective in helping people quit smoking	Strongly agree, Agree, Disagree and Strongly disagree
	My current knowledge is sufficient for helping parents to reduce SHS exposure to children	Strongly agree, Agree, Disagree and Strongly disagree
	My current knowledge is sufficient for helping patients to stop smoking	Strongly agree, Agree, Disagree and Strongly disagree
	I can assess a smoker's different stages of readiness to quit	Strongly agree, Agree, Disagree and Strongly disagree
Knowledge of SHS exposure	SHS causes sudden Infant Death Syndrome	Yes, No, and Don't know
	SHS causes adult lung cancer	Yes, No, and Don't know
	SHS causes adult heart disease	Yes, No, and Don't know
	SHS causes bronchitis	Yes, No, and Don't know
	SHS causes children's pulmonary disease	Yes, No, and Don't know
	SHS causes asthma in children	Yes, No, and Don't know
	SHS causes respiratory infections in children	Yes, No, and Don't know
	Breathing air in a room today where people smoked yesterday can harm the health of infants and children	Yes, No, and Don't know
	Paternal smoking increases the risk of lower respiratory tract illnesses such as pneumonia in exposed children	Yes, No, and Don't know

Copy Right Statement

These questions were used by the co-authors (Abdullah and Winickoff) in their earlier studies and agreed to publish the questionnaire in relation to the current publication.

© 2015 by the authors; licensee MDPI, Basel, Switzerland. This article is an open access article distributed under the terms and conditions of the Creative Commons Attribution license (http://creativecommons.org/licenses/by/4.0/).
